# Sociodemographic factors associated with physical activity and sedentary behavior in Brazilian adults living with type 1 diabetes: a cross-sectional study

**DOI:** 10.1590/1516-3180.2024.0215.R1.27112024

**Published:** 2025-06-06

**Authors:** Bruno Pereira de Moura, Bruna Priscila Colombo, Valter Paulo Neves Miranda, Isabella Toledo Caetano, Jeffer Eidi Sasaki, Paulo Roberto dos Santos Amorim

**Affiliations:** I Programa de Pós-Graduação em Ciências Médicas, Faculdade de Ciências Médicas, Universidade do Estado do Rio de Janeiro (UERJ), Rio de Janeiro (RJ), Brazil.; II Programa de Pós-Graduação em Saúde da Família, Faculdade de Ciências Médicas, Universidade do Estado do Rio de Janeiro (UERJ), Rio de Janeiro (RJ), Brazil.; III Instituto Federal de Educação, Ciência e Tecnologia Goiano (IF Goiano), Urutaí (GO), Brazil.; IV Departamento de Educação Física, Universidade Federal de Viçosa (UFV), Viçosa (MG), Brazil.; V Programa de Pós-Graduação em Educação Física, Universidade Federal do Triângulo Mineiro (UFTM), Uberaba (MG), Brazil.; VI Departament of Biomedical and Biotechnological Sciences, School of Medicine, Research Center on Motor Activities (CRAM), University of Catania, Catania, Italy.

**Keywords:** Diabetes mellitus, Accelerometry, Motor activity, Sedentary behavior, Sociodemographic factors, Socioeconomic factors, Actigraph, Type 1 diabetes, Sedentary lifestyle, Physical activity

## Abstract

**BACKGROUND:**

Physical activity (PA) and sedentary behavior (SB) are key determinants of health outcomes in individuals living with type 1 diabetes (T1D). However, the influence of sociodemographic and clinical factors on engagement in these behaviors is not yet well understood.

**OBJECTIVE:**

This study aimed to analyze the associations of sociodemographic factors, body mass index, and both the duration and age at diagnosis of diabetes with SB, light-intensity physical activity (LPA), and moderate-to-vigorous physical activity (MVPA) in Brazilian adults.

**DESIGN AND SETTING:**

A cross-sectional study was conducted at the Diabetes and Metabolism Service of a public secondary care unit in Rio de Janeiro.

**METHODS:**

One hundred adults diagnosed with T1D had their daily awake time spent in SB, LPA, and MVPA measured using triaxial accelerometers. Sociodemographic and clinical factors were assessed using questionnaires. Generalized Linear Models were used to analyze the relationships of these factors with SB, LPA, and MVPA.

**RESULTS:**

Significant associations were found between age, education level, and employment status with SB and LPA, but not with MVPA. On average, each additional year of age was associated with decreased time in SB and increased time in LPA. Higher education levels and unemployment were linked to more SB and less time in LPA.

**CONCLUSIONS:**

Age, education level, and employment status emerged as key sociodemographic predictors of SB and LPA in Brazilian adults living with T1D. These findings contribute to a better understanding of the sociodemographic determinants associated with SB and PA in individuals diagnosed with T1D.

## BACKGROUND

Physical activity (PA) and sedentary behavior (SB) are widely acknowledged as crucial determinants of health outcomes,^1^ particularly in individuals with chronic conditions such as type 1 diabetes (T1D).^2,3^ The interaction between these daily physical behaviors, which includes light-intensity physical activity (LPA), moderate-to-vigorous intensity physical activity (MVPA), and SB, significantly impacts glycemic control, cardiovascular risk, and overall well-being in this group of individuals.^3,4,5,6^ Notably, engagement in PA or SB extends beyond individual choice and is influenced by a range of sociodemographic and clinical factors.^7,8,9^

Within Brazil’s diverse skin color, culture, and socioeconomic context, it is essential to understand the factors that influence daily physical behaviors in adults living with T1D. Factors such as age, age at diabetes diagnosis, disease duration, and education level may affect an individual’s propensity towards PA or sedentary habits.^7^ Furthermore, sociodemographic characteristics such as gender, marital status, self-reported skin color, employment status, and socioeconomic status can either promote or hinder engagement in regular physical activities, potentially exacerbating health risks.^7–9^

Over the past decade, the increased use of accelerometers has enhanced our understanding of the relationship between SB, PA, and health outcomes.^10,11^ Despite the significance of this topic, there is a noticeable gap in the literature regarding the interplay of specific sociodemographic and clinical determinants of engagement in daily physical behaviors among Brazilian adults diagnosed with diabetes. Given Brazil’s diverse ethnic and socioeconomic composition, these factors may interact differently than in other regions, both within and outside the country. Understanding these determinants in the Brazilian context is crucial for developing targeted interventions and effective public policies, including health promotion and education strategies tailored to patients’ needs.

## OBJECTIVE

This study aimed to analyze the associations of sociodemographic factors, body mass index, and both the duration and age at diagnosis of diabetes with SB, LPA, and MVPA in Brazilian adults diagnosed with T1D.

## METHODS

### Study design and participants

This cross-sectional study was conducted between 2015 and 2016 in Rio de Janeiro, and included a convenience sample of 105 participants. The sample size calculation, estimated using G*Power Software (v.3.1.9.2),^12^ indicated that 89 participants were necessary to detect a minimum effect size of 0.2, assuming an alpha error of 5% and a power of 80%. All participants had a diagnosis of T1D and were randomly invited to participate while receiving care at the Diabetes and Metabolism Service of a public secondary care unit. The inclusion criteria required participants to be adults (aged 19 years or older) of any gender, diagnosed with T1D for a minimum of one year.^13^ All participants signed the consent form and completed all study procedures. Exclusion criteria included individuals who reported severe cardiovascular disease or any other comorbidities causing functional disability. The study adhered to the ethical principles outlined in the Declaration of Helsinki^14^ and received approval from the local Ethics Committee (CAAE: 30848814.0.0000.5259) on May 20, 2014.

### Data collection and analysis

#### Sociodemographic and clinical factors

Sociodemographic and clinical data were collected using a questionnaire covering age, age at diabetes diagnosis, disease duration, gender, education level, marital status, self-reported skin color, employment status, and socioeconomic status. Anthropometric evaluations included body weight (kg) and height (m). Body mass index was calculated by dividing weight (kg) by the square of height (m^2^). All measurements were conducted by a trained technician following the protocols established by Lohman et al.^15^ Participants were categorized according to the World Health Organization’s recommendations.^16^

Education level was determined based on the International Standard Classification of Education.^17^ For analysis, levels were grouped into three categories: primary education, secondary education, and undergraduate and graduate school. Marital status was classified into two groups: married and unmarried (single, widowed, and divorced).^18^ Self-reported skin color was categorized as white or non-white (black and brown). Participants reported their employment status at the time of the interview (employed or unemployed). Socioeconomic status was evaluated using the Brazilian Economic Classification Criteria.^19^ The six original categories (A, B1, B2, C1, C2, and D-E) were consolidated into two groups for this study: non-low (encompassing A, B1, and B2) and low (including C1, C2, and D-E).

#### Daily physical behaviors

Daily awake time spent in SB and PA was quantified using the ActiGraph™ wGT3X-BT triaxial accelerometer (ActiGraph LLC, Pensacola, Florida). This device records triaxial acceleration along the X, Y, and Z axes, with a sensitivity range of ± 8 g and a sampling frequency between 30 and 100 Hz.^20^ Each accelerometer was initialized via ActiLife^®^ software (v6.13.3) to capture data at a sampling frequency of 30 Hz.

Participants were instructed to wear the accelerometer on the right side of the hip, aligned with the axillary line of the iliac crest, secured by an elastic belt, for eight consecutive days, removing it only during sleep or water-based activities such as bathing or swimming.^11^ Detailed written instructions on device usage and researcher contact information were provided. Participants were encouraged to continue their regular daily routines. The accelerometer data were downloaded using ActiLife^®^ software and converted into 60-second epoch activity count data (counts/min). Activity counts were based on vector magnitude, and specific cutoff points categorized daily physical behaviors.^21,22^ The thresholds for SB, LPA, and MVPA were set at < 200 counts/min, 200–2690 counts/min, and ≥ 2691 counts/min, respectively.^21,22^ The data underwent a quality and compliance review after download.

Non-wear time was calculated using an algorithm devised by Choi et al.^23^ A valid day was defined as one with more than 600 minutes (10 hours) of wear time. Inclusion in the analysis required at least three valid days, including two weekdays and one weekend day.^11,23^ To minimize the Hawthorne effect,^24^ accelerometers recorded data from 00:00 hours on the second day to 23:59 hours on the seventh day, excluding data from the first and eighth days. Consequently, the analysis included up to six continuous days of data. Participants reported their typical wake-up and bedtimes on both weekdays and weekends via open-ended questions (“What time do you normally get up?” and “What time do you normally go to bed?”).^25^ These responses were used to estimate time in bed, which was treated as non-wear time. Periods of SB and PA lasting at least 10 minutes, with an allowance of up to 2 minutes for interruptions, were recognized. In the ActiLife^®^ software, the “ignore first sedentary break of each day” option was selected, and non-wear periods were excluded from the analysis.

### Statistical analysis

Descriptive statistics were employed to summarize and characterize the data, presented as means with standard deviation (SD) or as n (%). Generalized Linear Models, appropriate for cross-sectional studies, were used to assess the associations between sociodemographic and clinical factors and each type of daily physical behavior (SB, LPA, and MVPA). The distribution of outcome variables was examined using histograms and density plots. The normality of residuals was ascertained through Q-Q plot analysis, which only tested the primary effects of sociodemographic factors (age, gender, education level, marital status, self-reported skin color, employment status, and socioeconomic status) and clinical factors (age at diabetes diagnosis, disease duration, and body mass index), adjusted for daily awake time (min). The model with the lowest Akaike Information Criterion (AIC) was selected as the best fit.

The model that demonstrated the best fit for the SB and MVPA outcomes used the Gamma distribution with the identity link function and the Wald confidence interval method. For LPA, the Gaussian distribution with the identity link function and Wald confidence interval method was considered the most convergent model. All statistical analyses were performed using the open-source software Jamovi (Version 2.4.11),^26^ with a set significance level of 5%.

## RESULTS

### Studied Population

Of the 105 participants initially recruited, five (4.7%) were excluded due to non-compliance with the inclusion criteria. Consequently, the final sample for this cross-sectional study comprised 100 Brazilian adults living with T1D. The sociodemographic and clinical characteristics of the participants are detailed in Table 1.

**Table 1:** Descriptive analysis of sociodemographic and clinical characteristics of Brazilian adults living with type 1 diabetes (n = 100)

Characteristic	Mean ? SD or n (%)
Age (years)	37.1 ± 12.1
Age at diabetes diagnosis (years)	19.9 ± 9.8
Duration of Diabetes (years)	17.2 ± 10.2
Body mass index (kg/m^2^)	25.1 ± 4.8
Gender	
*Male*	41 (41)
*Female*	59 (59)
Education level	
*Primary education*	10 (10)
*Secondary education*	49 (49)
*Undergraduate and graduate school*	41 (41)
Marital status	
*Married*	36 (36)
*Non-married*	64 (64)
Self-reported skin color	
*White*	51 (51)
*Non-white*	49 (49)
Employment status	
*Employed*	60 (60)
*Unemployed*	40 (40)
Socioeconomic status	
*Low*	48 (48)
*Non-low*	52 (52)

SD: Standard deviation. The unmarried category comprises single, widowed, and divorced individuals. The non-white category comprises people whose self-reported skin color was black or brown. The non-low category refers to strata A, B1, and B2 of the Brazilian Economic Classification Criteria.

### Daily physical behaviors

Regarding accelerometer data, participants wore the devices for an average of 5.6 days, accumulating approximately 1,008 minutes (16.8 hours) of daily awake time, as detailed in Table 2. The range of accelerometer wear was 3 to 6 days, with 75% of participants wearing the device for 6 days. As a result, 94% of the data were collected from participants with at least 5 days of wear.

**Table 2:** Accelerometer-assessed sedentary behavior, light-intensity physical activity, and moderate-to-vigorous physical activity in Brazilian adults living with type 1 diabetes

Accelerometer data, mean ± SD	All	Female	Male
Daily awake time (min)	1008.1 ± 87	998.5 ± 83.8	1021.8 ± 90.6
Valid days	5.6 ± 0.7	5.6 ± 0.7	5.6 ± 0.6
SB (min/day)	526.7 ± 109.6	519.3 ± 110	537.5 ± 109.5
SB (%/day)	52.3 ± 10.1	51.9 ± 9.6	52.9 ± 10.8
LIPA (min/day)	434.7 ± 105.1	436.8 ± 100.2	431.6 ± 112.9
LIPA (%/day)	43.1 ± 9.4	43.8 ± 9.4	42.0 ± 9.5
MVPA (min/day)	46.6 ± 27.5	42.3 ± 22.5	52.7 ± 32.7
MVPA (%/day)	4.6 ± 2.6	4.2 ± 2.2	5.1 ± 3

No statistical difference was found for gender, significance level P < 0.05.

Brazilian adults living with diabetes allocated 52.3% of their daily awake time to SB, 43.1% to LPA, and only 4.6% to MVPA. This translates to an average of 526.7 min/day (8.8 hours/day) in SB, 434.7 min/day (7.2 hours/day) in LPA, and 46.6 min/day in MVPA.

On average, men had a daily awake time 23.3 minutes longer than women (1021.8 min vs. 998.5 min). However, no statistically significant gender differences were observed in SB, LPA, or MVPA.

**Figure 1:**
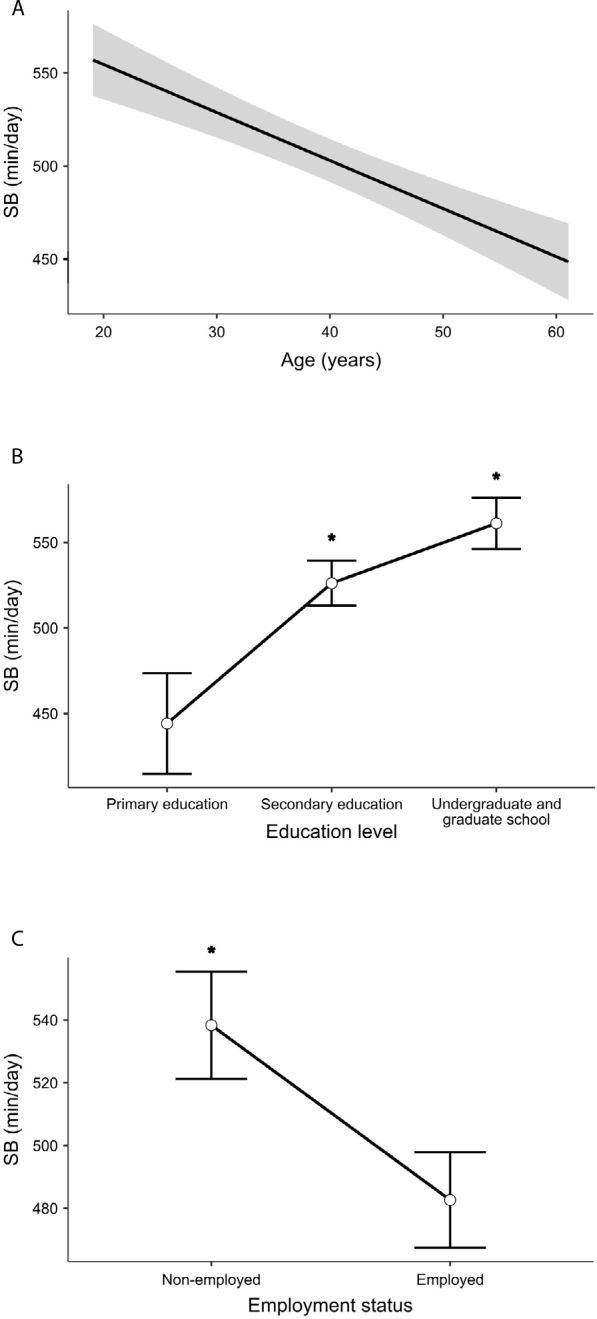
Associations of age, education level, and employment status with sedentary behavior in Brazilian adults living with type 1 diabetes.

**Figure 2:**
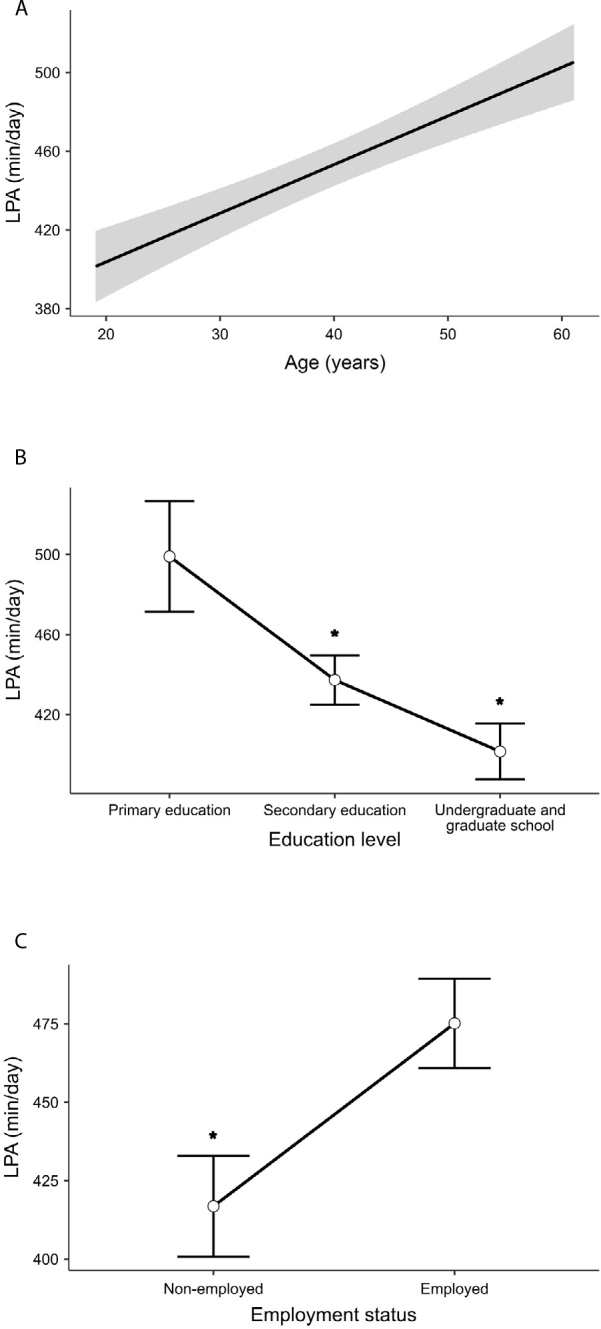
Associations of age, education level, and employment status with light-intensity physical activity in Brazilian adults living with type 1 diabetes.

Generalized Linear Models revealed a significant model only for SB (R^2^ = 0.35; P < 0.001) and LPA (R^2^ = 0.38; P < 0.001). Of all factors analyzed, only age, education level, and employment status showed statistically significant associations with SB and LPA (Table 3, Figures 1, 2). On average, for each additional year of age, Brazilian adults living with T1D exhibited a 2.6 min/day reduction in SB (β = −2.6, SE: 0.7; P < 0.001). Nonetheless, individuals with higher education levels and those in the unemployed group tended to be more sedentary (Figures 1b, 1c). Compared to individuals with primary education, those with secondary education spent an average of 74.5 more min/day in SB (β = 74.5, SE: 27.3; P = 0.006), and those with undergraduate and graduate education spent an average of 104.5 more min/day in SB (β = 104.5, SE: 28.9; P < 0.001). Regarding employment status, unemployed participants spent an average of 47.2 more min/day in SB compared to the employed group (β = 47.2, SE: 21.9; P = 0.032).

**Table 3:** Generalized Linear Models for sedentary behavior, light-intensity physical activity, and moderate-vigorous physical activity in Brazilian adults with type 1 diabetes

Factor	SB^a^	LPA^a^	MVPA^a^
	β (95% CI)	β (95% CI)	β (95% CI)
Age (years)	**-2.6** **(-4.1, -1.1)**	**2.5** **(1.0, 3.9)**	0.1 (-0.2, 0.5)
Education level			
*Primary education*	Reference	Reference	
*Secondary education*	**74.5** **(18.7, 126.1)**	**-61.7(-121.1, -2.2)**	
*Undergraduate and graduate school*	**104.5** **(45.9, 159.4)**	**-97.4** **(-158.5, -36.2)**	
Employment status			
*Employed*	Reference	Reference	
*Unemployed*	**47.2** **(5.1, 89.5)**	**-58.3** **(-99.5, -17.1)**	

SB = Sedentary behavior; LPA = Light-intensity physical activity; MVPA = Moderate-to-vigorous physical activity.

^a^ Adjusted for daily waking time (min). Significant associations (P < 0.05) are indicated in bold.

a) Association between age and sedentary behavior (SB), accounting for educational level and employment status, and adjusted for daily awake time (β = −2.6; SE: 0.7; P < 0.001); b) Association between educational level and SB, accounting for age and employment status, and adjusted for daily awake time (Secondary education vs. Primary education [reference]: β = 74.5; SE = 27.3; P = 0.006, and Undergraduate and graduate school vs. Primary education [reference]: β = 104.5; SE = 28.9; P = < 0.001); c) Association between employment status and SB, accounting for age and educational level, and adjusted for daily awake time (Unemployed: β = 47.2; SE: 21.9; P = 0.032); SE: Standard error. * Statistically significant difference compared to the reference group.

a) Association between age and light-intensity physical activity (LPA), accounting for educational level and employment status, and adjusted for daily awake time (β = 2.5; SE: 0.7; P < 0.001); b) Association between educational level and LPA, accounting for age and employment status, and adjusted for daily awake time (Secondary education vs. Primary education [reference]: β = −61.7; SE = 30.3; P = 0.042, and Undergraduate and graduate school vs. Primary education [reference]: β = −97.4; SE = 31.2; P = 0.002); c) Association between employment status and LPA, accounting for age and educational level, and adjusted for daily awake time (Unemployed: β = −58.3; SE: 21.0; P = 0.006); SE: Standard error. * Statistically significant difference compared to the reference group.

In terms of LPA, each additional year of age was linked with an increase of 2.4 min/day in LPA (β = 2.5, SE: 0.7; P < 0.001) (Figure 2a). In contrast, individuals with higher education levels and those in the unemployed group had shorter daily engagement times (Figure 2b, 2c). When compared to the primary education group, those with secondary education spent an average of 61.7 fewer min/day in LPA (β = −61.7, SE: 30.3; P = 0.042), and those with undergraduate or graduate education spent an average of 97.4 fewer min/day in LPA (β = −97.4, SE: 31.2; P = 0.002). Unemployed participants had an average of 58.3 fewer min/day in LPA compared to their employed counterparts (β = −58.3, SE: 21.0; P = 0.006).

**Figure 3:**
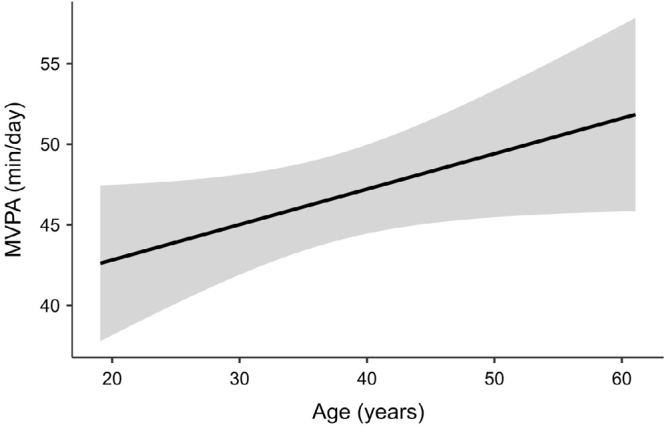
Associations of age with moderate-to-vigorous physical activity in Brazilian adults living with type 1 diabetes.

Regarding MVPA, the best-adjusted model included only the age factor (R^2^ = 0.05). However, no sociodemographic or clinical factors were significantly associated with MVPA (Figure 3).

Association between age and moderate-to-vigorous physical activity (MVPA), adjusted for daily awake time (β = 0.1; SE: 0.2; P = 0.410) SE: Standard error.

## DISCUSSION

To the best of our knowledge, this is the first study to investigate the sociodemographic and clinical factors associated with the objective measurement of SB, LPA, and MVPA among Brazilian adults living with T1D. The best-adjusted model for the data in this study indicated that only age, education level, and employment status emerged as predictors for SB and LPA, with no sociodemographic or clinical factors being associated with MVPA.

Understanding the impact of sociodemographic factors on daily physical behaviors is a critical step towards developing more effective public health initiatives to promote PA for disease prevention or to mitigate the adverse effects of diabetes and its comorbidities.^7,9,27^ Our findings reveal that with each additional year of age, the adults evaluated exhibited an average decrease of 2.6 min/day in SB, while LPA showed an increase of 2.4 min/day. This outcome contradicts traditional reports that link aging with increased SB.^28,29^ A study conducted among Brazilian adults diagnosed with type 2 diabetes (T2D) revealed that aging was associated with increased daily time spent in sedentary behavior and reduced time spent in MVPA.^29^ These findings may be correlated with the etiological distinction between T1D and T2D. The influence of age on SB and LPA may be a specific characteristic of young adults affected by this disease, as 57% of individuals in this study were in the 20–39 year age range, and T1D generally includes a younger age at diagnosis (< 35 years).^13^ Data from this sample indicate that although young adults diagnosed with T1D have high rates of SB, they modify their behavioral patterns over time, reducing time spent in SB and increasing daily time engaged in LPA. Therefore, this result suggests that young adults living with T1D can assimilate physical activities as an effective non-pharmacological strategy for lifelong diabetes management.

Studies^7,9^ of individuals diagnosed with diabetes have reported higher PA levels associated with younger age. However, the use of self-report instruments for PA assessment in these studies may have influenced the results.^30,31,32^ Although initial analysis of the effect size of age for both SB and LPA may suggest minimal clinical impact, considering the outcome variable’s unit of measurement (min/day), there is a reduction of 949 minutes (15.8 hours) per year in SB and an increase of 876 minutes (14.6 hours) in LPA. This evidence suggests that LPAs could be an effective and feasible alternative to MVPA for promoting the health of this population.

Regarding education level, our study found that individuals with higher education levels tended to be more sedentary and, consequently, spent less time on LPA. This finding aligns with that of another study of patients living with T1D,^7^ which showed that higher education levels were correlated with more time spent in SB. The sedentary nature of study-related activities, which require prolonged sitting, limits opportunities for active behaviors, thereby increasing daily SB levels. Compensatory strategies to reduce sitting time should be considered,^33^ such as taking breaks to stand or walk every 2 hours or using an unstable gym ball instead of a conventional chair.^34,35^

Employment status was also a significant predictor of SB and LPA. Employed participants reported less time in SB and more time in LPA compared to the unemployed group. Depending on job characteristics, employed individuals may have more opportunities for physical activities such as LPAs than unemployed individuals. Studies^7,8^ of different populations living with T1D have identified similar effects of employment status on SB and PA. However, methodological differences in SB and PA assessment prevent direct comparison with the results of the present study.

In our study, no significant associations were found between sociodemographic or clinical factors and MVPA. Notably, Brazilian adults diagnosed with T1D spent most of their daily awake time in SB, approximately 43% in LPA, and only a small fraction in MVPA (4.6%). The high prevalence of SB, combined with diabetes-related metabolic disorders, presents a concerning scenario as SB is strongly associated with increased cardiovascular disease risk and mortality.^36^ Although high-intensity physical activities like MVPA offer substantial cardiometabolic benefits,^37^ engaging in such activities can be challenging for individuals with chronic illnesses, especially those living with T1D, due to risks such as hypoglycemia.^5,38,39^ Therefore, the low levels of MVPA observed in this sample, and their maintenance throughout life, may be associated with a fear of hypoglycemia.^38,39^ Recently, a systematic review with meta-analysis^40^ investigated the relationship between LPA, cardiometabolic health, and mortality. The findings of this review suggest that LPA can contribute to improved health and reduced mortality risk.

Therefore, although a published guideline^27^ indicates that there is insufficient evidence to determine the influence of sociodemographic factors on PA and SB in people living with diabetes, our study contributes new insights, highlighting the impact of age, education level, and employment status on SB and LPA.

This study, however, has certain limitations. As a cross-sectional study with a convenience sample, its results should be interpreted within this specific context. While cross-sectional studies are effective for exploring potential correlates of SB and PA, they do not permit causal inferences. In this study, the protocol for using accelerometers 24 hours a day was not implemented, which precluded direct analysis of sleep duration. Unfortunately, at the time of data collection, the specific professions of participants who identified as employed were not recorded. The absence of this information makes it impossible to detail the relationship between the type of occupational activity and levels of daily physical activity. Among the strengths of our study is the objective assessment of SB and PA in daily routines using reliable wGT3X-BT accelerometers. Furthermore, most of our data were obtained from participants with substantial accelerometer usage (5–6 days), ensuring the reliability of our findings.

## CONCLUSION

In conclusion, our findings indicate that age, education level, and employment status are significant predictors of SB and LPA, but not of MVPA, in Brazilian adults living with T1D. The reduction in sedentary time and the increase in LPA with age, along with the influence of higher education levels and employment status on increased SB, highlight the need for targeted interventions to promote healthier activity patterns in this population. This study contributes to a better understanding of the complex interplay of sociodemographic determinants influencing SB and PA in Brazilian adults living with diabetes and lays the groundwork for future public health initiatives tailored to the needs of this population.
